# Impact of 45S5-Bioactive Glass on Chondrocytes in Knee Osteoarthritis—In Vitro Study Exploring Cellular Responses

**DOI:** 10.3390/jfb16090339

**Published:** 2025-09-09

**Authors:** Max Marinescu, Sébastien Hagmann, Jörg Fellenberg, Elena Tripel, Simone Gantz, Ravikumar Mayakrishnan, Aldo R. Boccaccini, Tobias Renkawitz, Babak Moradi, Fabian Westhauser, Hadrian Platzer

**Affiliations:** 1 Department of Orthopaedics, Heidelberg University Hospital, 69118 Heidelberg, Germany; 2 Orthopedic Research Center, Kiel University, 24118 Kiel, Germany; 3 Institute of Biomaterials, University of Erlangen-Nuremberg, 91085 Erlangen, Germany; 4 Department of Orthopaedics, University of Regensburg, Asklepios Klinikum Bad Abbach, 93077 Bad Abbach, Germany; 5 Department of Orthopedics and Trauma Surgery, University Medical Center Schleswig-Holstein, Campus Kiel, 24105 Kiel, Germany

**Keywords:** 45S5-bioactive glass, osteoarthritis, chondrocytes, matrix metalloproteinases, cytokines

## Abstract

Osteoarthritis (OA), the most common joint disease, is marked by cartilage degradation and chronic inflammation. While 45S5-bioactive glass (45S5-BG) is well-established in bone regeneration and has been suggested to exert immunomodulatory effects, its impact on OA chondrocytes remains largely unexplored. Therefore, this in vitro study investigated the effects of 45S5-BG microparticles (0.125 mg/mL) on chondrocytes derived from OA patients, evaluating its therapeutic potential in OA. Chondrocytes were cultured with or without 45S5-BG for 1 and 7 days. Gene expression of cartilage markers, cytokines, matrix metalloproteinases (MMPs), and toll-like receptors (TLRs) was analyzed by qPCR. Protein levels were assessed by ELISA. 45S5-BG stimulation significantly altered chondrocyte activity, inducing upregulation of IL-6, IL-1β, TNF-α, MMP-1/-3/-13, and TLR4. Expression of ACAN and COL2A1 was reduced, while COL10A1—a marker of chondrocyte hypertrophy—was significantly increased at day 1. These findings show a catabolic and pro-inflammatory shift in chondrocyte phenotype upon 45S5-BG exposure, showing no therapeutic benefit of 45S5-BG on OA chondrocytes. However, considering the pronounced effects on chondrocyte activity and the well-established bioactivity and biocompatibility of 45S5-BG, our findings suggest that modified BG formulations could be developed to enhance chondroprotective and anti-inflammatory properties, warranting further investigation in co-culture and in vivo models.

## 1. Introduction

Osteoarthritis (OA) is the most prevalent joint disease globally, and is responsible for physical and social constraints, causing a substantial loss in quality of life [1]. Once regarded as merely a degenerative wear and tear condition, OA is now commonly recognized as a multifactorial disease [2,3]. Beyond the mechanical components, mounting evidence underscores the critical role of biochemical alterations, particularly inflammatory processes which cause an imbalance between anabolic and catabolic factors, in advancing cartilage degradation and therefore disease progression [4,5]. Among these, synovial inflammation has emerged as a defining feature in symptomatic patients with OA and is recognized as an autonomous contributor to OA etiology [6]. It leads to infiltration of various inflammatory cell types, including macrophages and T-cell subsets [7], precipitating, together with fibroblasts, the release of inflammatory mediators and matrix-degrading enzymes, thereby exacerbating cartilage degradation [2,4]. Beyond synovial tissue derived inflammation, cartilage itself, long regarded as a passive target, is now recognized as an active and major contributor to joint inflammation. In OA, articular chondrocytes undergo phenotypic changes characterized by a shift from anabolic homeostasis to a catabolic and hypertrophic state [2,4]. This drives the gradual loss of cartilage tissue from the joint surface and is one of the defining pathological features of osteoarthritis [1,8,9]. Despite expanding understanding of inflammatory mechanisms in OA, the absence of regulatory-approved disease-modifying osteoarthritis drugs (DMOADs) underscores the imperative for innovative treatment solutions [10,11].

Among various biomaterials explored for regenerative and immunomodulatory applications, bioactive glasses (BGs) have gained growing attention. 45S5-bioactive glass (45S5-BG), composed of 45% SiO_2_, 24.5% Na_2_O, 24.5% CaO, and 6% P_2_O_5_ by weight, was originally developed for bone regeneration and is still widely used both in research and in the clinic [12,13,14]. 45S5-BG has since expanded its spectrum of applications, ranging from wound healing enhancement to antimicrobial efficacy and oncological interventions [15,16,17,18,19,20,21]. Importantly, the incorporation of substances with anti-inflammatory properties or biologically active ions into bioactive glasses has been shown to augment their therapeutic effectiveness [22,23,24,25]. Recent investigations have demonstrated the capacity of 45S5-BG to modulate inflammatory processes, exhibiting a propensity towards an anti-inflammatory trajectory [23,25,26,27,28,29]. Given the central role of inflammation in OA pathogenesis and the emerging immunomodulatory properties of 45S5-BG, the question arises of whether bioactive glasses could influence inflammatory processes in OA. Previously, it was demonstrated that 45S5-BG microparticles can modulate the production of matrix metalloproteinases (MMPs) and cytokines in synovial cells from OA patients [30]. However, the effects of 45S5-BG on OA chondrocytes have not been investigated. Supporting the potential of 45S5-BG to influence chondrocyte behavior and highlighting the relevance of our study, pro-chondrogenic effects have already been reported in studies using non-OA chondrocyte phenotypes [31,32,33]. Given that cellular behavior varies depending on phenotype, this in vitro study was designed to assess, for the first time, the direct impact of pure 45S5-BG microparticles on human articular chondrocytes derived from OA patients. Understanding this interaction is critical, as the cellular response to the unmodified material provides the basis for the rational development of functionalized bioactive glass formulations or scaffold-integrated systems targeting cartilage protection and regeneration in OA.

Considering the existing literature, we analyzed both the gene expression and protein secretion profiles of key mediators involved in OA pathogenesis. Specifically, we examined MMPs (MMP-1/-2/-3/-9/-13) [34,35,36,37], disintegrins and metalloproteases with thrombospondin motif (ADAMTS) family members (ADAMTS-4/-5), pro-inflammatory cytokines (interleukin (IL) IL-1β/-6, tumor necrosis factor alpha (TNF-α)), and toll-like receptors (TLR) TLR-2 and TLR-4 [38,39,40,41,42,43,44,45]. Additionally, we examined key markers of hyaline cartilage matrix in OA: aggrecan (ACAN), collagen type I alpha 1 (COL1A1), collagen type II alpha 1 (COL2A1), and collagen type X alpha 1 (COL10A1) [46,47,48].

## 2. Materials and Methods

### 2.1. Study Design Overview

**Figure 1:**
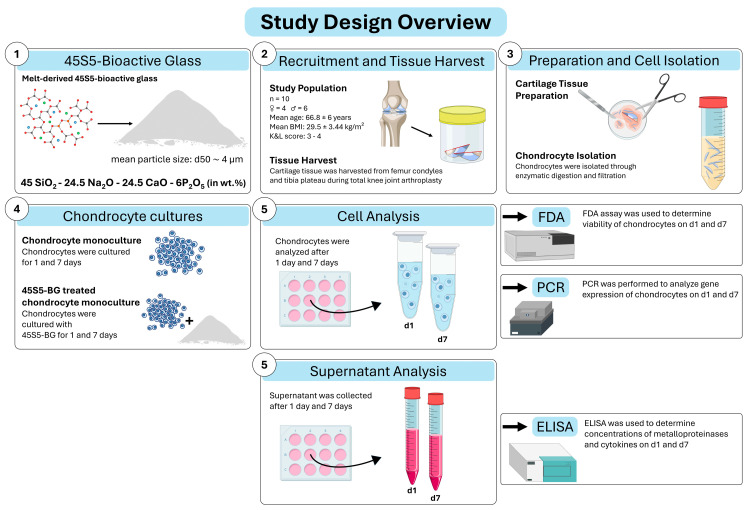
Study design overview. Abbreviations: 45S5-bioactive glass (45S5-BG), Fluorescein diacetate (FDA), polymerase chain reaction (PCR), enzyme-linked immunosorbent assay (ELISA), percentage by weight (wt.%), day 1/7 (d1/7).

In short, 45S5-bioactive glass was sourced and 10 patients with advanced knee OA were recruited. Cartilage tissue was harvested, and chondrocytes were isolated through instrumental preparation and enzymatic digestion. Chondrocyte monocultures with and without 45S5-BG were cultivated for 1 and 7 days. At each time point, cell viability was assessed using fluorescein diacetate (FDA) staining, and gene expression was analyzed by polymerase chain reaction (PCR) analysis. Additionally, protein concentrations were analyzed in culture supernatants by enzyme-linked immunosorbent assays (ELISAs) (Figure 1).

### 2.2. Study Population

The demographic characteristics of the study population are summarized in Table 1. A total of 10 patients participated in the study, comprising four females and six males, with a mean age of 66.8 ± 6.1 years and a mean body mass index (BMI) of 29.5 ± 3.4 kg/m^2^. All patients were diagnosed with advanced knee osteoarthritis (OA) based on the criteria established by the American College of Rheumatology, with a Kellgren and Lawrence score (K&L score) ranging from 3 to 4. All the patients underwent total knee arthroplasty at the Department of Orthopaedics at Heidelberg University Hospital. Prior to surgery, none of the patients demonstrated signs of systemic inflammation, as assessed by both clinical examination and blood analysis (C reactive protein (CRP) levels and leukocyte counts). Additionally, none of the participants reported recent trauma, use of disease-modifying antirheumatic drugs (DMARDs), intra-articular knee injections within the three months preceding surgery, or regular use of nonsteroidal anti-inflammatory drugs (NSAIDs). This study was approved by the ethics committee of the University of Heidelberg (approval code: S 603/2021) and informed consent was obtained from all participants before enrollment in the study.

### 2.3. Sample Collection

Cartilage tissue was harvested during total knee arthroplasty surgery from femur condyles and parts of tibial plateaus and submerged in phosphate-buffered saline (PBS) in sterile containers. The samples were immediately prepared further.

### 2.4. Bioactive Glass

In this study, we used melt-derived 45S5-bioactive glass powder (Schott Vitryxx, Mainz, Germany) with a composition of 45% SiO_2_, 24.5% Na_2_O, 24.5% CaO, and 6% P_2_O_5_ in weight. The physicochemical characteristics of this standard 45S5-BG, including particle morphology and ion release behavior, have been extensively described in the literature [49,50]. According to the manufacturer and independent research, the glass powder exhibits a mean particle size of d_50_~4 μm (µm) with d_95_ ≤ 20 µm. Scanning electron microscopy imaging typically shows angular particle morphology [49,50].

### 2.5. Chondrocyte Isolation

Cartilage tissue was separated with surgical scalpels and washed with phosphate-buffered saline (PBS), after which the cartilage was cut into fine slices with surgical scissors. These cartilage pieces were combined with Dulbecco’s modified Eagle’s medium (DMEM) containing 10% fetal calf serum (FCS) (Biochrom, Berlin, Germany) and 1% penicillin–streptomycin (P/S) (10 mg/mL, Biochrom, Berlin, Germany), and digested with 20 mg/mL of collagenase B and 1 mg/mL of hyaluronidase for approximately 12 hours (h) at 37 degrees Celsius (°C) while rotating. After enzymatic digestion, the cell suspension was filtered through a 40 µm filter and washed with PBS.

### 2.6. Cell Expansion and Cell Culture

Isolated chondrocytes were counted using a Neubauer hemocytometer and cultured as expansion cultures with culture medium (89% DMEM, 10% FCS, 1% P/S) at 37 °C with 5% CO_2_ in 175 cm^2^ culture flasks (Thermo Fisher Scientific, Waltham, MA, USA). The medium was changed after 24 h and 2–3 days. Non-adherent chondrocytes and cell debris were removed through rinsing with PBS. After five days of incubation, chondrocytes were extracted from the incubation flasks through treatment with trypsin (Bio&SELL GmbH, Feucht, Germany) for 3 min at 37 °C.

Chondrocytes were cultured as monolayers with and without the presence of 45S5-BG in 12-well plates containing 5 × 10^4^ cells per well at 37 °C and 5% CO_2_. This cell density format has been successfully used previously [7]. A total of 0.125 mg/mL 45S5-BG was established as the highest non-toxic concentration for chondrocytes (see Section 2.7). On day 1 (d1) and day 7 (d7), the culture supernatant was collected and stored at −80 °C for enzyme-linked immunosorbent assay (ELISA) analysis (see Section 2.9). Additionally, on d1 and d7, cells were analyzed for cell viability using fluorescein diacetate assays (see Section 2.7), and RNA was extracted from chondrocytes for gene expression analysis (see Section 2.8).

### 2.7. Cell Viability Assay

Cell viability was assessed using fluorescein diacetate (Sigma–Aldrich, St. Louis, MO, USA) as described previously [30,51]. In short, viable cells hydrolyze FDA into a green, fluorescent product which accumulates intracellularly. The fluorescence intensity correlates with cell viability and reflects the viable cell number. Briefly, for the FDA viability assay, the culture supernatant was removed, and chondrocytes were washed with PBS, stained with 0.002 mg/mL of FDA, and incubated for 10 minutes (min) at 37 °C in the dark. Afterwards, excess FDA was removed by rinsing with PBS. The cells were then lysed with 1% Triton X-100 buffer (Sigma–Aldrich, St. Louis, MO, USA) and fluorescence signal intensity was quantified at 535 nanometers (nm) for emission and 485 nm for excitation utilizing a Wallac 1420 Victor 2 microplate reader (PerkinElmer, Waltham, MA, USA).

FDA cell viability staining was also used in the initial experiments to determine 45S5-BG toxicity towards chondrocytes. Isolated chondrocytes were cultivated as monolayers in the presence of 45S5-bioactive glass at concentrations ranging from 0.0625 mg/mL to 1 mg/mL. After 1 and 7 days, FDA staining was used to assess cell viability.

### 2.8. RNA Extraction, cDNA Synthesis, Real-Time Quantitative PCR

RNA extraction and purification from cells were conducted on days 1 and 7 using the PureLink RNA Mini Kit (Invitrogen, Darmstadt, Germany) according to the manufacturer’s instructions. RNA quantity and quality were assessed using a NanoDrop ND-1000 spectrophotometer (PeqLab; Erlangen, Germany). For cDNA synthesis, the Biozym cDNA Synthesis Kit (Biozym Scientific GmbH, Hessisch Oldendorf, Germany) was used in accordance with the manufacturer’s instructions. A total of 250 ng of RNA was combined in a 20 µL reaction mixture, which was incubated for 30 min at 42 °C and afterwards for 10 min at 85 °C. The resulting cDNA was diluted 1:5 with 10 mM Tricine (Carl Roth, Karlsruhe, Germany) and stored at −20 °C.

Subsequently, 2 μL of cDNA was subjected to qPCR analysis using PrimaQuant CYBR QPCR master mix (Steinbrenner Laborsysteme, Wiesenbach, Germany). The thermal cycling program executed on a LineGene 9600 thermal cycler (Bior Technologies, Hangzhou, China) included preheating at 95 °C for 5 min, followed by 40 cycles of denaturation at 95 °C for 15 seconds (s), annealing at 58 °C for 20 s, extension at 72 °C for 20 s, and a melting curve analysis. The expression of ribosomal protein S13 (RPS13) in the corresponding samples was used for normalization. This study analyzed the gene expression levels of aggrecan (ACAN), collagen type I/II/X alpha 1 (COL1A1/2A1/10A1), MMP-1/-2/-3/-9/-13, ADAMTS-4/-5, IL-6/-1β, TNF-α, and TLR-2/-4. All the primers used are listed in Supplementary Table S1.

### 2.9. ELISA

MMP and cytokine concentrations were measured in culture supernatants according to the manufacturer’s instructions using enzyme-linked immunosorbent assays (ELISAs) for MMP-1 (DY901B; R&D Systems, Minneapolis, MN, USA), MMP-2 (RAB0365; Sigma-Aldrich, St. Louis, MO, USA), MMP-3 (DY513; R&D Systems, Minneapolis, MN, USA), MMP-9 (DY911; R&D Systems, Minneapolis, MN, USA), MMP-13 (RAB0364; Sigma-Aldrich, St. Louis, MO, USA), ADAMTS-4 (RAB1020; Sigma-Aldrich, St. Louis, MO, USA), ADAMTS-5 (DY2198; R&D Systems, Minneapolis, MN, USA), and IL-6 (DY206; R&D Systems, Minneapolis, MN, USA). Photometric measurements were performed using an Autobio PHOmo Microplate Reader (Autobio Diagnostics Co., Ltd., Zhengzhou, China).

The minimum detectable doses of the ELISA kits were declared by the respective manufacturers as follows: MMP-1, 62.5 picograms (pg) per mL; MMP-2, 3500 pg/mL; MMP-3, 31.2 pg/mL; MMP-9, 31.2 pg/mL; MMP-13, 6.0 pg/mL; ADAMTS-4, 120.0 pg/mL; ADAMTS-5, 125.0 pg/mL; and IL-6, 9.4 pg/mL. Concentrations below these thresholds were considered undetectable.

### 2.10. Statistical Analysis

PCR data were evaluated using the delta–delta Ct (ΔΔCt) method and normalized to the housekeeping gene RPS13. ELISA results were normalized by calculating the ratio of each analyte concentration to the corresponding FDA fluorescence value from the same well. This normalization approach was performed to improve data accuracy by controlling for subtle differences in cell viability—even at non-toxic 45S5-BG concentrations. Normality was assessed using the Kolmogorov–Smirnov test. For normally distributed data, paired t-tests were performed, and the Wilcoxon signed-rank test was applied for non-normally distributed data. Statistical significance was set at *p* < 0.05. *p*-values are marked as follows: * *p* < 0.05, ** *p* < 0.01, *** *p* < 0.001. Statistical analysis was performed using SPSS version 29.0.2.0 (IBM Corp., Mannheim, Germany). Graphs were constructed using GraphPad Prism, version 9 (GraphPad Software, La Jolla, CA, USA).

## 3. Results

### 3.1. The Impact of 45S5-BG on Chondrocyte Viability

**Figure 2:**
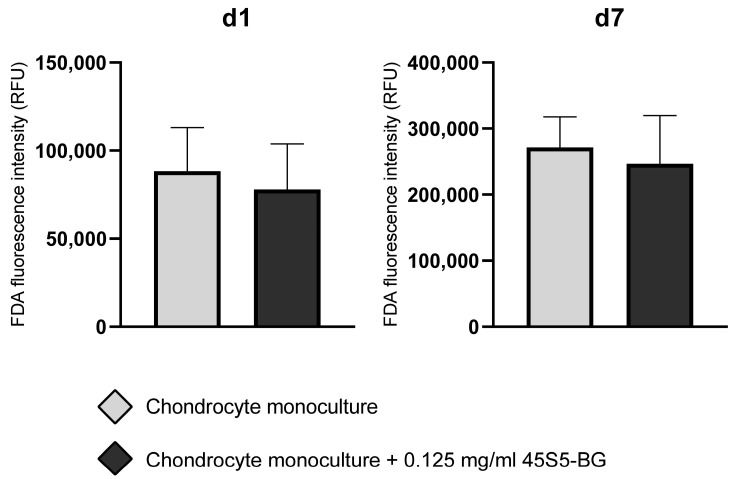
Fluorescein diacetate (FDA) fluorescence intensity of chondrocytes cultured with and without 45S5-bioactive glass (45S5-BG). Columns represent the mean ± standard deviation of relative fluorescence unit (RFU) values at day 1 (d1) and day 7 (d7) of cell culture (n =10). Data represent absolute fluorescence intensities as directly measured by the plate reader. No normalization was applied.

Initial experiments were conducted to determine the cytotoxic threshold of 45S5-BG microparticles on human articular chondrocytes from OA patients. Chondrocyte monocultures were exposed to increasing concentrations of 45S5-BG ranging from 0.0625 mg/mL to 1 mg/mL. FDA viability assays revealed a concentration-dependent reduction in cell viability, with notably decreased fluorescence signals observed at concentrations exceeding 0.125 mg/mL. At and below 0.125 mg/mL, fluorescence intensity remained stable across multiple replicates, indicating preserved cell viability. No cytotoxic effect was detected at this concentration at either day 1 or day 7 (n = 10). Therefore, 0.125 mg/mL was determined to be the highest non-toxic concentration under the given experimental conditions and was selected for subsequent analyses (Figure 2).

### 3.2. The Impact of 45S5-BG on Chondrocyte Gene Expression

**Figure 3:**
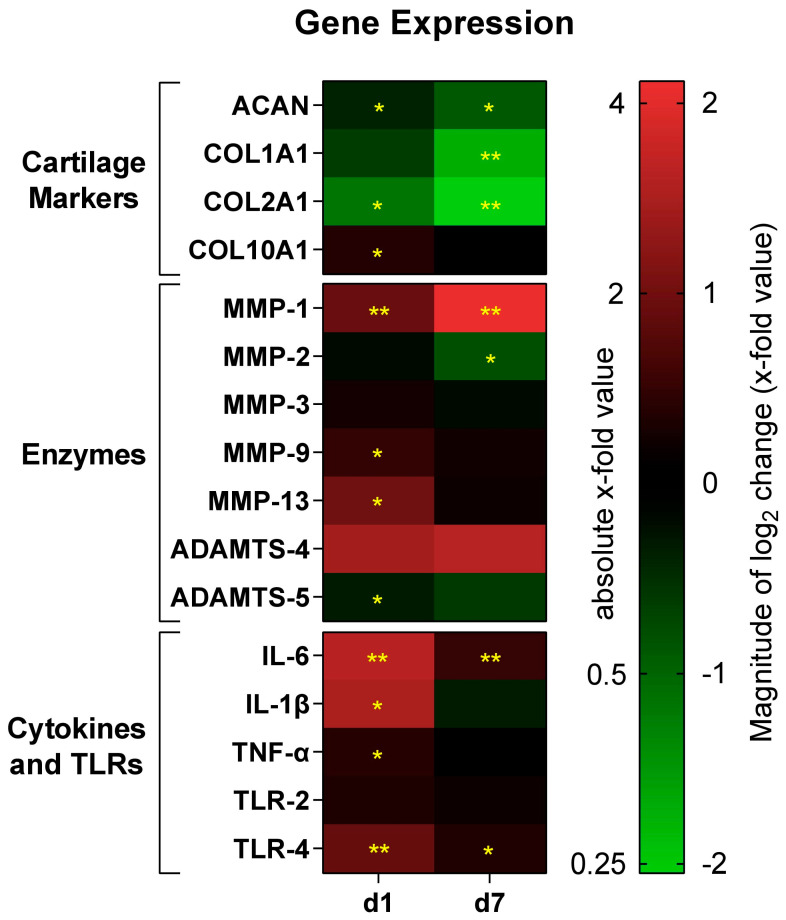
Heatmap of gene expression. Expression data are shown as absolute and log_2_ fold changes in gene expression of osteoarthritic (OA) chondrocytes treated with 45S5-bioactive glass compared to untreated OA chondrocytes at day 1 (d1) and day 7 (d7). Values represent relative expression changes determined by qPCR using the ΔΔCt method. Upregulated genes are displayed in red tones, and downregulated genes in green tones, with color intensity corresponding to the magnitude of the fold change. Significant differences are indicated by asterisks: * *p* < 0.05, ** *p* < 0.01.

**Figure 4:**
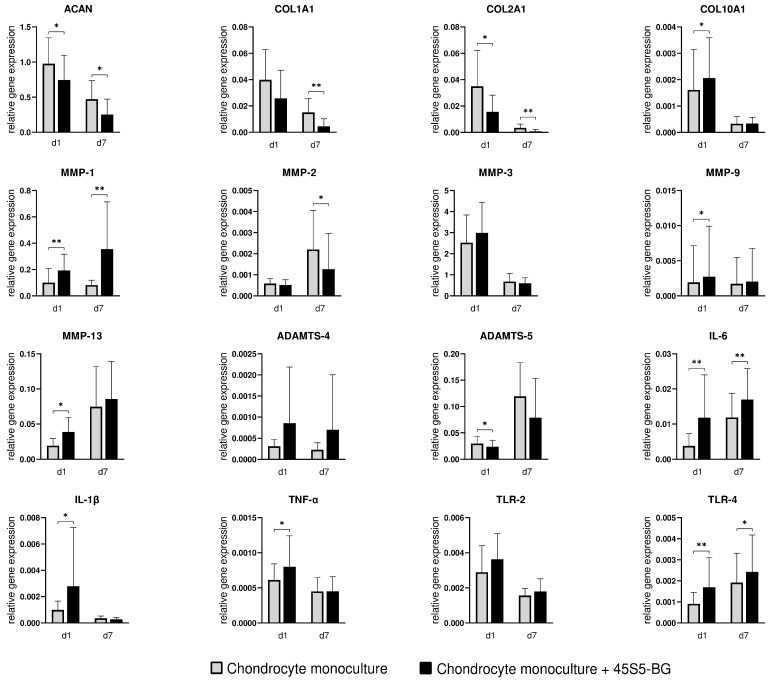
Gene expression profile of osteoarthritic (OA) chondrocytes with and without 45S5-bioactive glass (45S5-BG) treatment at day 1 (d1) and day 7 (d7). Mean and standard deviation of relative expression levels, normalized to RPS13 and calculated using the ΔΔCt method (qPCR), are shown. Significant differences are indicated by asterisks: * *p* < 0.05, ** *p* < 0.01.

To investigate the molecular response of chondrocytes to 45S5-BG microparticles, the gene expression profile of key cartilage markers (ACAN, COL1A1, COL2A1, COL10A1), matrix-degrading enzymes (MMP-1, MMP-2, MMP-3, MMP-9, MMP-13, ADAMTS-4, ADAMTS-5), and pro-inflammatory mediators and immune receptors (IL-6, IL-1β, TNF-α, TLR-2, TLR-4) was analyzed using qPCR (Figure 3 and Figure 4).

#### 3.2.1. Cartilage Markers

Treatment with 45S5-BG significantly reduced the expression of ACAN and COL2A1 at both d1 and d7 (fold change of 0.76 and 0.54, respectively, for ACAN and 0.45 and 0.24, respectively, for COL2A1). COL1A1 expression was also significantly reduced at d7. Conversely, COL10A1 was significantly upregulated in chondrocyte monocultures in the presence of 45S5-BG at d1.

#### 3.2.2. Enzymes

Expression of MMP-1 was significantly increased at both d1 and d7 (fold change of 1.91 and 4.33 respectively), while expression of MMP-9 and MMP-13 was upregulated at d1. In contrast, MMP 2 expression was significantly downregulated at d7 and ADAMTS-5 showed reduced expression at d1. No significant changes were observed for MMP-3 or ADAMTS-4.

#### 3.2.3. Inflammatory Cytokines and Toll-like Receptors

An inflammatory gene expression signature was observed in 45S5-BG-treated chondrocytes. IL-6 and TLR-4 were significantly upregulated at both d1 and d7 (fold change of 3.10 and 1.43, respectively, for IL-6 and 1.87 and 1.27, respectively, for TLR-4). IL-1β and TNF-α also showed a significant increase in expression at d1. No significant difference was found for TLR-2 expression.

To provide a comprehensive overview of gene expression changes, a heatmap was used to visualize log_2_-transformed fold changes across all the investigated genes (Figure 3). This format allows for immediate pattern recognition across different pathways and time points. To complement this overview, individual bar graphs are presented (Figure 4).

### 3.3. The Impact of 45S5-BG on Chondrocyte MMP and Cytokine Production

To assess whether the transcriptional changes observed in 45S5-BG-treated chondrocytes translated into altered protein secretion, ELISA analyses of culture supernatants were performed for key matrix metalloproteinases (MMP-1, MMP-2, MMP-3, MMP-9, MMP-13), aggrecanases (ADAMTS-4, ADAMTS-5), and the inflammatory cytokine IL-6. Although no significant cytotoxicity was observed at the applied 45S5-BG concentration, ELISA values were normalized to FDA fluorescence to account for potential subtle differences in cell viability or metabolic state. This approach enables a more accurate per-cell interpretation of secreted protein levels, rather than a crude per-well measurement. It thus helps to disentangle true biological effects from mere differences in viable cell number or metabolic competence.

Among the measured analytes, MMP-3 was consistently detected at the highest concentrations, followed by MMP-1, MMP-13, and IL-6. In contrast, MMP-2, MMP-9, ADAMTS-4, and ADAMTS-5 were below the ELISA detection levels, indicating minimal or no translation to the protein level and secretion under the culture conditions used in this study.

**Figure 5:**
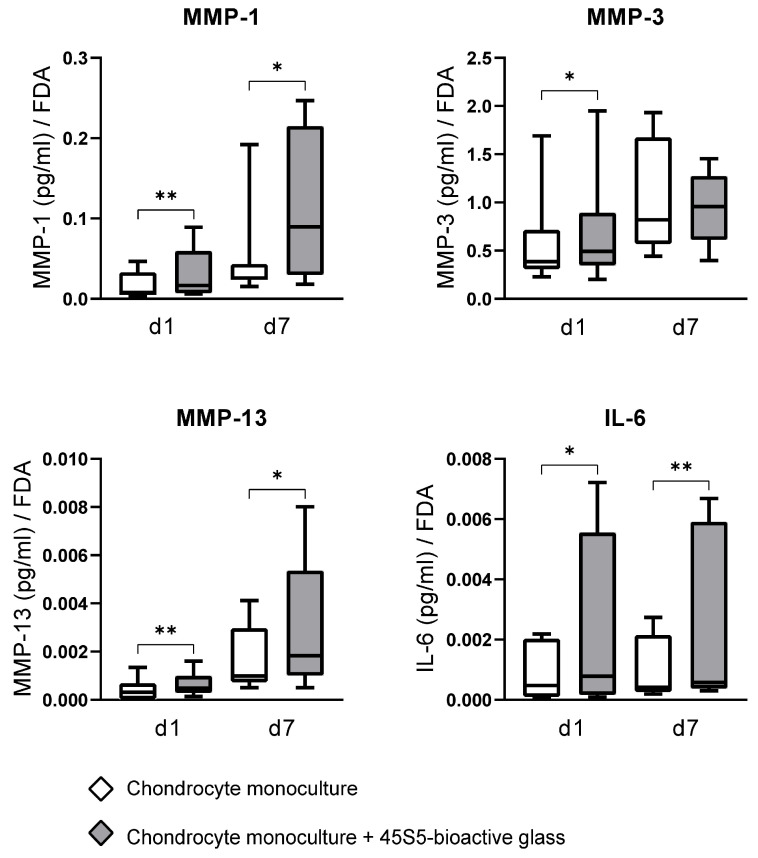
Quantitative analysis of matrix metalloproteinase (MMP) and Interleukin-6 (IL-6) concentrations in the supernatant of chondrocyte monocultures with and without 45S5-bioactive glass (45S5-BG). Enzyme-linked immunosorbent assays (ELISAs) were used to analyze MMP and IL-6 concentrations in culture supernatants on day 1 (d1) and day 7 (d7). Quotients of analyte concentration to the corresponding FDA value (fluorescence intensity) were calculated. The mean and standard deviation are plotted. Significant differences are indicated by asterisks: * *p* < 0.05 ** *p* < 0.01.

Chondrocytes cultured with 45S5-BG microparticles showed significantly elevated levels of MMP-1 (p_d1_ = 0.007, p_d7_ = 0.022), MMP-13 (p_d1_ = 0.005, p_d7_ = 0.021), and IL-6 (p_d1_ = 0.013, p_d7_ = 0.008) in the culture supernatant at both d1 and d7 compared to untreated chondrocyte monocultures. MMP-3 secretion was also significantly increased at d1 (*p* = 0.037, Figure 5).

## 4. Discussion

This in vitro study demonstrates that pure and unmodified 45S5-BG microparticles have a significant impact on the activity of chondrocytes derived from patients with knee OA in vitro. Preliminary experiments indicated dose-dependent cytotoxicity of 45S5-BG to OA chondrocytes, with 0.125 mg/mL identified as the highest non-toxic concentration under our experimental conditions. Gene expression and protein secretion analyses revealed a largely consistent pattern of increased inflammatory and catabolic activity in OA chondrocytes treated with 45S5-BG compared to untreated cells.

To our knowledge, this is the first study to investigate the direct effects of unmodified 45S5-BG on primary human OA chondrocytes as basis for the rational development of functionalized bioactive glass formulations or scaffold-integrated systems targeting cartilage protection and regeneration in OA. While previous reports have suggested immunomodulatory or even pro-chondrogenic effects of bioactive glass in non-OA models [23,25,26,27,28,29,31,32,33], our findings emphasize the importance of disease-specific material evaluation. The analytes selected in this study reflect hallmark mediators of cartilage metabolism and inflammation in OA, allowing for an integrated assessment of both transcriptional and functional responses to 45S5-BG exposure.

### 4.1. The Impact of 45S5-BG on Cartilage Marker Expression

45S5-BG-treated chondrocytes exhibited a decrease in the expression of ACAN, COL1A1, and COL2A1, while an increase in the expression of COL10A1 was observed. These changes in gene expression suggest a catabolic effect of pure 45S5-BG on hyaline cartilage. Aggrecan (ACAN) is a central proteoglycan in healthy articular cartilage and contributes to its load-bearing properties through its highly charged glycosaminoglycan chains, which attract and retain water and form large aggregates by binding to hyaluronan and linking proteins, thereby creating a hydrated network that resists compression [46,52]. Collagen type I alpha 1 (COL1A1) is a major structural protein found in most connective tissues that provides tensile strength and structural integrity. Its presence in articular cartilage is typically associated with fibrotic tissues, such as fibrocartilage, which is considered inferior to hyaline cartilage for articular function [53]. Type II alpha 1 collagen (COL2A1) is the predominant collagen in hyaline cartilage and is primarily produced by chondrocytes. It forms the main structural framework by creating a fibrillar network and plays a central role in maintaining cartilage integrity and metabolism [53]. Collagen type X alpha 1 (COL10A1) is produced by hypertrophic chondrocytes and found in calcified articular zones and around cartilaginous lesions. It also provides structural integrity while being involved in mineralization processes [53,54]. The downregulation of ACAN and COL2A1, key markers of healthy hyaline cartilage [46,53], without compensatory upregulation of COL1A1, suggests general suppression of extracellular matrix-related gene activity by treatment with 45S5-BG. The increase in COL10A1 expression indicates a shift towards hypertrophic differentiation, suggesting an osteoarthritic stimulating effect. Together, these findings indicate that the pure 45S5-BG microparticles used in this study do not promote the maintenance or regeneration of a stable hyaline cartilage phenotype.

### 4.2. The Impact of 45S5-BG on Metalloproteinases

We further analyzed the impact of 45S5-BG on the expression and secretion of key matrix-degrading enzymes, including MMP-1, MMP-2, MMP-3, MMP-9, MMP-13, ADAMTS-4, and ADAMTS-5. These enzymes are central to cartilage matrix turnover and are closely linked to OA pathogenesis [34]. While 45S5-BG had no measurable effect on the secretion of gelatinases MMP-2/-9 or the aggrecanases ADAMTS-4/-5 in this study, it significantly changed the expression and secretion of collagenases (MMP-1 and MMP-13) and stromelysin-1 (MMP-3) in OA chondrocytes.

### 4.3. Collagenases (MMP-1, MMP-13) and Stromelysin-1 (MMP-3)

Regarding gene expression levels, treatment with 45S5-BG resulted in significant upregulation of MMP-1 and MMP-13, two key collagenases involved in OA pathogenesis. This was accompanied by increased protein secretion into the culture supernatant, as confirmed by ELISA. MMP-1 (interstitial collagenase) can digest collagenous structures (collagen types I, II and III) [34,55], while MMP-13 (collagenase 3) degrades collagen fibrils, particularly type II, and aggrecan, and is primarily produced by chondrocytes [34,56]. Therefore, the results from this study suggests a degrative response and catabolic shift in chondrocytes upon exposure to 45S5-BG. Notably, MMP-3 (stromelysin-1), which degrades non-collagenous matrix components such as proteoglycans, fibronectin, and laminin, and also activates procollagenases [34,56,57,58,59], showed the highest absolute concentration in all chondrocyte monocultures in this study, aligning with previous studies on isolated synovial membrane cells treated with 45S5-BG [30] or without bioactive glass treatment [7]. This recurring pattern shows that MMP-3 is released across multiple joint-resident cell types in OA and suggests that 45S5-BG can robustly stimulate MMP-3, potentially contributing to cartilage matrix destabilization [34,60].

### 4.4. The Impact of 45S5-BG on Inflammatory Cytokines and the Toll-like Receptor Profile

IL-6 is a pro-inflammatory cytokine that induces MMP and ADAMTS activity [39,61] and plays a central role as catabolic mediator in many diseases, including OA [4,39,62]. In this study, chondrocytes exposed to 45S5-BG exhibited increased IL-6 gene expression at both day 1 and day 7, accompanied by elevated IL-6 protein levels in culture supernatant, indicating a BG-induced pro-inflammatory response in OA chondrocytes. In addition, gene expression of IL-1β and TNF-α was upregulated at day 1 following 45S5-BG exposure. Both cytokines are key inflammatory mediators in OA pathogenesis, involved in cartilage degradation through the induction of MMPs, such as MMP-1, MMP-3, and MMP-13, and the suppression of collagen type II [34,38,39,42].

Regarding toll-like receptors, in this study, TLR-4 expression was significantly increased at both time points in 45S5-BG-treated chondrocytes, while TLR-2 expression remained unchanged. Toll-like receptors act as pattern recognition receptors (PRRs) and play an important role in innate immune responses. Their activation in OA has been implicated in pro-inflammatory and catabolic signaling through pathways such as NF-κB [63,64,65]. The selective upregulation of TLR-4 expression, without a corresponding change in TLR-2, may reflect partial activation of PRR-mediated pathways in response to 45S5-BG exposure. However, whether this change represents a stress response, or the initiation of a more persistent receptor profile switch, remains unclear. This warrants further investigation, particularly as TLR-2 may play a more prominent role in OA-associated inflammation [66,67].

Taken together, our findings suggest that pure 45S5-BG microparticles provoke a predominantly inflammatory response in OA chondrocytes, reflected by upregulation of key inflammatory cytokines and enzymes and changes in the PRR profile. Whether this response reflects transient cellular stress, or the onset of a more permanent catabolic chondrocyte phenotype, remains unclear. The findings from this in vitro study indicate that unmodified 45S5-BG microparticles alone are not suitable for OA therapy. Considering previous findings reporting the anabolic effects of 45S5-BG in non-OA chondrocytes [33], our results reveal the importance of disease-specific biomaterial evaluation. The chondrocyte phenotype itself may play a critical role in determining the cellular response to bioactive glass exposure.

Importantly, bioactive glasses are characterized by their tunable composition, which has already enabled the development of numerous modified bioactive glass formulations with expanded therapeutic profiles. For instance, incorporation of biologically active ions or bioactive compounds has been shown to modulate immune responses and enhance regenerative effects in various settings [22,23,24,25]. Our study provides a foundational reference by establishing that pure 45S5-BG microparticles directly alter the activity of human OA chondrocytes in vitro. This provides a valuable basis for further research exploring whether and how 45S5-BG compositional modifications can redirect this response toward a cartilage-preserving or even pro-anabolic chondrocyte phenotype. For instance, the addition of copper into bioactive glass–ceramic scaffolds has been shown to increase chondrocyte proliferation and maturation while promoting an anti-inflammatory environment [68]. Barium-doped bioactive glass significantly increased IL-10 and reduced IL-6 and TNF-α levels in vitro compared to 45S5-BG, exhibiting anti-inflammatory effects [27]. Similarly, the introduction of strontium or magnesium ions into 45S5-BG has been shown to promote cell viability and antimicrobial effects [25], while zinc incorporation into bioactive glasses has demonstrated anti-inflammatory activity [69]. Therefore, future studies should investigate the effects of such or related modifications of 45S5-BG on human OA chondrocytes, ideally using co-culture or in vivo models that more closely mimic the complex joint microenvironment.

Some limitations of this study should be acknowledged. We observed that gene expression of OA chondrocytes changed between day 1 and day 7 in untreated monocultures, indicating an intrinsic shift during in vitro culture. This effect is in agreement with previous findings showing that primary human articular OA chondrocytes progressively lose their disease-specific gene expression profile during monolayer culture, including reduced expression of ACAN and COL2 over time [70]. These changes suggest that by day 7, cultured chondrocytes in this study may have partially drifted from their original OA-specific phenotype, while day 1 likely reflects the initial in vivo state of freshly isolated chondrocytes better. Importantly, however, the validity of our comparisons between 45S5-BG-treated and untreated groups at each respective time point remains unaffected. The observed catabolic and pro-inflammatory responses to 45S5-BG exposure thus remain robust within this experimental framework. Nonetheless, future experiments should use three-dimensional culture systems or in vivo models to preserve the disease-relevant phenotype of OA chondrocytes more effectively over time. It should also be noted that not all transcriptional changes detected at the mRNA level translated into corresponding alterations in protein secretion. Such discrepancies are commonly observed in cell culture experiments and may occur due to post-transcriptional regulation, differential protein stability, or secretion kinetics. In addition, the detection sensitivity of the ELISAs employed may have limited the detection of low-abundance proteins. In this study, patient comorbidities such as obesity, diabetes, or cardiovascular disease, which may influence cartilage metabolism, were not systematically recorded and taken into account. Future studies should integrate these parameters to enable a more precise interpretation of cellular responses to bioactive glasses. It should also be noted that the physicochemical properties of 45S5-bioactive glass, including particle size, surface area, and morphology, influence its dissolution behavior and subsequent biological responses. In the present study, however, as only a single type of bioactive glass was examined, a direct link between specific material characteristics and the observed biological effects cannot be conclusively established within the scope of this study. Future studies directly comparing bioactive glass formulations with systematically varied particle characteristics will be required to clarify these relationships in detail. Furthermore, this study was designed to investigate the effects of 45S5-bioactive glass microparticles on chondrocytes from OA patients in an isolated in vitro setting. While this approach allows for controlled analysis, it does not fully replicate the more complex interactions of the joint microenvironment. Moreover, no long-term cultures were performed, so potential delayed or long-lasting effects on chondrocyte activity under the influence of 45S5-BG microparticles need to be further evaluated in follow-up studies.

## 5. Conclusions

This in vitro study demonstrates that 45S5-bioactive glass influences both anabolic and catabolic activity in chondrocytes derived from patients with knee osteoarthritis. The upregulation of key inflammatory cytokines and matrix-degrading enzymes, alongside the downregulation of hyaline cartilage markers, suggests that pure 45S5-BG microparticles exert a predominantly cartilage destructive and pro-inflammatory effect, promoting a shift towards a hypertrophic and catabolic chondrocyte phenotype. Therefore, the findings of this in vitro study do not support a beneficial therapeutic effect of 45S5-BG on OA chondrocytes. However, given the compositional adaptability of bioactive glasses and the pronounced effects of 45S5-BG on chondrocyte gene expression and protein secretion, there is considerable potential to mitigate these largely undesired effects by exploiting the capability of BGs to be modified by the incorporation of a great variety of biologically active ions. Future studies should investigate such modified formulations and their therapeutic potential in the context of osteoarthritis, preferably in more complex and in vivo models.

## Figures and Tables

**Table 1:** The demographic and clinical parameters of the study population are shown. Data are displayed as mean ± standard deviation and interquartile range (IQR) or as number (%). BMI = body mass index.

	Total Study Population (n = 10)
Sex, n (%)	
Female	4 (40%)
Male	6 (60%)
Age (years), mean ± SD; IQR	66.8 ± 6.1; 10.8
BMI (kg/m^2^), mean ± SD; IQR	29.5 ± 3.4; 6.4
Leukocytes (cells/nL), mean ± SD; IQR	6.4 ± 1.1; 1.5
C-reactive protein (mg/L), mean ± SD; IQR	1.8 ± 3.3; 3.5

## Data Availability

The original contributions presented in the study are included in the article; further inquiries can be directed to the corresponding author.

## Supplementary Materials

The following supporting information can be downloaded at https://www.mdpi.com/article/10.3390/jfb16090339/s1. Table S1: Primers used for qPCR analyses.

## Abbreviations

The following abbreviations are used in this manuscript:

45S5-BG	45S5-bioactive glass
ΔΔCt	Delta–delta Ct
µm	Micrometers
°C	Celsius
ACAN	Aggrecan
ADAMTS	A disintegrin and metalloprotease with thrombospondin motifs
BMI	Body mass index
cDNA	Complementary deoxyribonucleic acid
cm^2^	Centimeter squared
COL	Collagen
CO_2_	Carbon dioxide
CRP	C-reactive protein
d1/7	Day 1/7
DMARDs	Disease-modifying antirheumatic drugs
DMEM	Dulbecco’s modified Eagle’s medium
DMOAD	Disease-modifying osteoarthritis drug
ELISA	Enzyme-linked immunosorbent assay
FCS	Fetal calf serum
FDA	Fluorescein diacetate
h	Hours
IL	Interleukin
K&L	Kellgren and Lawrence
mg	Milligrams
mL	Milliliters
MMP	Matrix metalloproteinase
NF-κB	Nuclear factor kappa-light-chain-enhancer of activated B cells
nm	Nanometers
NSAIDs	Nonsteroidal anti-inflammatory drugs
OA	Osteoarthritis
P/S	Penicillin–Streptomycin
PBS	Phosphate-buffered saline
pg	Picograms
PRR	Pattern recognition receptor
qPCR	Quantitative polymerase chain reaction
RNA	Ribonucleic acid
RPS13	Ribosomal protein S13
s	Seconds
TLR	Toll-like receptor
TNF	Tumor necrosis factor
