# Supplementary material for: Impact of 45S5-Bioactive Glass on Chondrocytes in Knee Osteoarthritis—In Vitro Study Exploring Cellular Responses
Source: J Funct Biomater. 2025 Sep 9;16(9):339. doi: 10.3390/jfb16090339 (PMC12470832; doi:10.3390/jfb16090339)
Supplement: Supplementary file 1 [file jfb-16-00339-s001.zip › jfb-3843765-supplementary.pdf]

Supplementary Table S1. Primers used for qPCR analyses

Gene	Forward primer (5´- 3´)	Reverse primer (5´- 3´)
**RPS13**	GGTTGAAGTTGACATCTGACGA	CTTGTGCAACACCATGTGAAT
**ACAN**	TCTGTCAGGCAAATCTGGGATGGT	ATGCCACTTGGTAGGCCACT
**COL1A1**	CAGGCTGGTGTGATGGGATT	GGGCCTTGTTCACCTCTCTC
**COL2A1**	GCTCCTGCCGTTTCGCTG	ATTATACCTCTGCCCATCCTGC
**COL10A1**	AAAGGCCCACTACCCAACAC	GTGGACCAGGAGTACCTTGC
**MMP-1**	CATGCTTTTCAACCAGGCCC	GGGTACATCAAAGCCCCGAT
**MMP-2**	GCTACGATGGAGGCGCTAAT	GGGCAGCCATAGAAGGTGTT
**MMP-3**	GTTTCCCTCCAACCGTGAGG	GCTATTTGCTTGGGAAAGCCT
**MMP-9**	CGACGTCTTCCAGTACCGAG	GTTGGTCCCAGTGGGGATTT
**MMP-13**	AGGAGCATGGCGACTTCTAC	AGACCTAAGGAGTGGCCGAA
**ADAMTS-4**	CATCCTACGCCGGAAGAGTC	TCTTGTCATCTGCCACCACC
**ADAMTS-5**	AAAGGGGAGAATCTGCCTGC	CCAAGATCCCCAGTTGCCAT
**IL-1β**	CGGCCACATTTGGTTCTAAGA	AGGGAAGCGGTTGCTCATC
**IL-6**	AGTGAGGAACAAGCCAGAGC	AGCTGCGCAGAATGAGATGA
**TNF-α**	ACTTTGGAGTGATCGGCCC	CATTGGCCAGGAGGGCATT
**TLR-2**	CTTCACTCAGGAGCAGCAAGCA	ACACCAGTGCTGTCCTGTGACA
**TLR-4**	AAAATCCCCGACAACCTCCC	TGTCTGGATTTCACACCTGGA

Agarose gel electrophoresis was used to control for primer specificity and efficacy.
